# Sales promotion by wholesalers affects general practitioners’ prescription behaviours in Japan

**DOI:** 10.1080/20016689.2018.1424474

**Published:** 2018-01-18

**Authors:** Hirohisa Shimura

**Affiliations:** ^a^ Healthcare Research Center, Crecon Research and Consulting Inc., Tokyo, Japan

**Keywords:** Japan, Japanese pharmaceutical market, prescription behaviour, wholesalers, sales function, marketing, promotion

## Abstract

**Background**: One method for promoting drugs in Japan has been utilizing wholesalers for promotion; however, the effectiveness of the sales promotion has been brought into question.

**Methods**: A total of 74,552 responses were collected from an internet survey of 511 prescribing doctors in hospitals with less than 19 beds, which recalled the visits by wholesalers’ sales representatives (MS) in 2014. Each assessed the degree to which MS and/or sales representatives from a pharmaceutical company (MR) influenced a decision to prescribe each drug. The responses were analysed using the chi-square test and Goodman-Kruskal’s gamma to evaluate the association between MS calls and doctors’ prescription orders.

**Results**: Results showed a significant effect of the MS calls on doctors’ behaviours in terms of new drug prescriptions and subsequent behaviours. The results by therapeutic category showed a similar strong influence of the joint calls on new prescriptions on some therapeutic classes. The MS calls significantly influenced doctors to maintain and increase the prescription volume (p < 0.01).

**Conclusion**: This paper demonstrates that sales promotion on the part of MSs and MRs adds value to the prescription decisions. Moreover, results suggest that MSs enhance prescription outcomes in competitive therapeutic categories.

## Introduction

The aging Japanese population has resulted in a steady growth of the Japanese pharmaceutical market [1]. As an illustration, in 2013, seniors accounted for 25% of Japan’s population [2]. Of all national medical care expenditures in Japan in 2002, 49.3% was spent on citizens aged 65 or more. This figure increased to 55.6% by 2011 [3], and is expected to continue to grow [4]. To address the challenges associated with an aging population, the Japanese government has implemented several initiatives, including the promotion of generic drugs by incentivizing pharmacies [5], as well as an increase in the consumption tax from 5% to 8% in April of 2014. The government implemented this tax increase to cover the rising social welfare costs linked to Japan’s aging population.

The government has implemented several measures to respond to population decline, including promoting the Community-based Integrated Care System by 2025. The system provides healthcare, long-term care, preventive long-term care, housing, and livelihood support in communities with a geographic size of a junior high school district [6]. After 2025 – the year in which the integrated care system will be implemented – healthcare providers will be divided such that they will operate either in a hospital or as part of the care system. Hospitals will be responsible for acute treatment of patients, and prescribing doctors in hospitals with fewer than 19 beds will serve the community care system. Currently, the Japanese pharmaceutical companies tend to focus much of their sales efforts on prescribing doctors in hospitals; the introduction of the community care system may cause the management of these companies to reconsider the way they allocate resources for sales.

Among pharmaceutical sales channels in Japan, pharmacies represent 53.7% of the market, hospitals account for 27.2% of the market, while prescribing doctors in hospitals with fewer than 19 beds or primary care doctors (hereafter simply called ‘doctors’) account for 18.4% [7]. Note that, in this paper, prices refer to actual transaction prices, rather than to official reimbursement prices by the Japanese government (NHI price), and sales also refer to the actual transaction amounts between wholesalers and medical institutions based on the actual transaction price. The Japanese prescription system is unique. In the past, prescription and dispensing were conducted at medical institutions. On the contrary, today most drugs are dispensed at medical institutions and prescribed at pharmacies. This practice was promoted by the Japanese government to make medical practitioners and pharmacists share duties in each specialisation to improve the quality of medical treatments [8]. Although the practice of separation between prescribing and dispensing has grown from 30.5% in 1998 to 68.7% in 2014 [9], pharmacists cannot replace generics without doctors’ consent. Given their influence over pharmacists, it is critical to engage in sales promotion targeted at doctors. Because there are no ethical means for pharmaceutical drug manufacturers to distribute their products to institutions that sell them, wholesalers are integral to the dissemination of pharmaceuticals in Japan [10].

In addition to distributing the pharmaceuticals themselves, Japanese pharmaceutical wholesalers have two important functions: price negotiation and sales promotion [11]. Medical sales representatives (MSs) are primarily responsible for these latter functions, and do so in their targeting of small hospitals and primary care markets in which pharmaceutical sales representatives (MRs) do not operate. In accordance with industry customs (as designated by ethical operations with respect to pharmaceuticals), MRs cannot negotiate the invoice price of a drug. The MSs can act as agents in this regard.

As the productivity of the pharmaceutical industry’s R&D has declined [12], companies have turned to several cost containment strategies. Among these strategies is sales promotion, the practice of which is often questioned in developed countries, including Japan [13]. In Japan, wholesalers engage in sales promotion and price negotiations in return for a distribution fee, rebates, and/or allowances [14]. The complexity of this system has resulted in ongoing arguments among pharmaceutical companies regarding the proper evaluation of the fees wholesalers collect. Despite this ongoing debate, few researchers have addressed the issue. Measuring the effectiveness with which wholesalers engage in sales promotions serves to clarify the needs of the wholesalers’ sales promotion functions and improve the efficiency with which pharmaceutical companies allocate resources within their own marketing functions and those practiced by wholesalers [15].

Many previous studies on the determinants of MR promotional effectiveness have focused on cases in the USA and UK [16–19]. Few, however, have explored this issue in the Japanese context. Many pharmaceutical companies have been forced to adopt new models for promoting and selling pharmaceuticals in a difficult environment [13]. Chressanthis et al. [16] determined that, given the limited access to physicians, MRs tend to focus on areas where they can have the greatest impact on sales, while others have contended that brand prescriptions add to the cost of care with little or no added value [17]. Janko and colleagues [18] further found that family physicians appreciate the scientific content and objectivity of information provided by MRs. Although aggressive marketing of pharmaceuticals can cause doctors to prescribe medicines on the basis of information about other scientific evidence, more than 75% of physicians found MRs’ visits to be valuable in learning about new drugs. Moreover, about 33% of physicians refer to MRs when deciding whether to prescribe a new drug [19]. Although it remains difficult to accurately measure the effectiveness of MR visits, it is nonetheless necessary to gauge the degree to which MRs and MSs are effective in promoting products in Japan.

To address the issues outlined above, this study investigates whether wholesalers effectively perform their sales promotion functions. More specifically, the degree to which MSs influence doctors’ tendencies to select certain drugs to be prescribed to patients was measured. The study further nuances these findings by delineating the effect of the MSs’ influence in terms of therapeutic categories and prescription outcomes.

Because there is no extant research on the effectiveness of MS sales promotion on doctors’ prescription decisions in Japan, past work has not been able to address the issue. In addition, the uniqueness of the Japanese context – which dictates that wholesalers can promote pharmaceutical products – yields a number of uncertainties about the effectiveness of MS in their capacity as product promoters. Therefore, this paper aims to evaluate the effect of MSs on doctors’ prescription behaviours by examining 1) the MS calls influence on doctors’ prescription habits in the absence of MR calls, and 2) whether MS visits amplify the influence of MRs on doctors’ prescription decisions. By investigating these two possibilities, it is possible to evaluate the effectiveness of the sales promotion function of Japanese wholesalers. This would also detect possible improvements in the MR activities, as they are related to pharmaceutical companies’ objectives (particularly with respect to market presence). As such, this study evaluates two hypotheses: 1) in the absence of a call by an MR (No MS co-visits), a call by an MS influences a doctor’s prescription decision and 2) MS visits (MS co-visits) amplify the influence of MRs on a doctor’s prescription decisions. To provide more detailed results, these hypotheses are also evaluated in terms of specific prescription outcomes and therapeutic categories.

## Materials and methods

The Minister of Health, Labour, and Welfare (MHLW) indicates that, when data were collected, there were 95,349 doctors working in hospitals and clinics with less than 19 beds [20]. Crecon Research & Consulting (Crecon), a consulting firm that performs empirical analyses of the healthcare industry, built a random sample of 95,349 doctors in hospitals with less than 19 beds to conduct a survey. Overall, the survey company randomly selected 1,234 doctors, 511 of which recalled the visits made by MSs. Of these, 511, 415 responded to the survey in 2014. Each doctor registered every MS visit resulting in drug prescription and was asked about the respective influences of MR and MS. A total of 74,552 responses, or cases in which doctors recalled the visits, were stored in a database called MS Call®. There is the possibility that all prescribed drugs were not recalled or recorded by doctors. These 74,552 responses were utilized for the analysis. The database exhibits a sampling error of 2.78% with a 95% confidence level. There are two possible types of MS visits: 1) MS alone (No MR co-visit), and 2) MR and MS joint visit (MR co-visit), with the database recording responses from MS visits. Each respondent assessed the degree to which an MS or MR influenced their decision to prescribe each drug on a three-point Likert scale ranging from 1 (strongly influenced) to 3 (no influence). If no MR called the respondent, then he/she could indicate as such (marked 4 on the scale). Doctors were prompted to make such an assessment in four distinct contexts (Table 1): when he/she 1) prescribed a new drug (New volume), 2) continued the use of a drug at the same prescription volume (Same volume), 3) continued the use of a drug at a higher prescription volume (Increasing volume), or 4) continued the use of a drug at a lower prescription volume (Decreasing volume).

**Table 1.** Descriptive statistics related to MS influence on doctor prescription decisions.

	New Volume			Same Volume			Additional Volume		
	Total	(MR co-visits)	(No MR co-visits)	Total	(MR co-visits)	(No MR co-visits)	Total	(MR co-visits)	(No MR co-visits)
Antidiabetic agents	742	698	44	5,996	5,218	778	1,421	932	489
Antihypertensive agents	453	401	52	6,132	5,087	1,045	1,306	921	385
Allergic drugs	260	213	47	3,963	3,213	750	775	505	270
Agents affecting metabolism	306	247	59	3,400	2,893	507	610	401	209
Peptic ulcer agents	122	77	45	2,489	1,879	610	495	320	175
Hyperlipidemia agents	150	123	27	2,358	1,835	523	559	386	173
Central nervous system agents	184	158	26	2,003	1,633	370	424	305	119
Ophthalmic agents	243	195	48	1,973	1,590	383	365	234	131
Respiratory agents	199	189	10	1,382	1,198	184	387	269	118
Vaccines	141	89	52	1,610	1,101	509	179	103	76
Total	5,527	4,595	932	56,799	44,508	12,291	11,255	7,410	3,845

Note: ‘New volume’ indicates that a doctor prescribed a new drug, ‘Same volume’ indicates that a prescribing doctor continued the use of a drug at the same prescription volume, ‘Increasing volume’ indicates that a prescribing doctor increased the prescription volume of a drug, while ‘Decreasing volume’ indicates that a prescribing doctor decreased the prescription volume. ‘MR co-visit’ indicates that MR and MS jointly visited a prescribing doctor, while ‘No MR co-visits’ indicates only MS visited a prescribing doctor. Therapeutic categories were based on the MHLW classification.

Note that it is very common that the MS visits doctors to deliver the actual drugs and drug information, and negotiate invoice price at the same time. The MS often makes an appointment for the MR with doctors in order to promote information about the drug, including usage and safety [14].

Doctors reported the effects of MS visits on their prescription decisions based on their memory of the visit every day through a specified website. Specifically, the doctor reported a ‘1ʹ if he/she was strongly influenced by an MR to prescribe a given pharmaceutical drug, a ‘2ʹ if he/she was influenced (though not strongly) by an MR, or a ‘3ʹ if he/she was not influenced. Participants were also given the option to indicate that no MR had called them. In this case, the value of the scale would be 4. It was important to offer a response option that accounted for MRs that did make a visit, because it is a Japanese business custom for pharmaceutical delegates to call wholesalers to promote their drugs rather than have an MR visiting small hospitals and clinics. In these cases, MS and MR would visit the doctor if he/she required further consultation. This scale was applied to drugs in the top 10 categories as identified by the MHLW. In this way, it was possible to evaluate the differences in prescription practices based on specific pharmaceutical therapies as categorised by the MHLW.

The McNemar’s chi-square test and Goodman-Kruskal’s gamma [21] were utilized to evaluate the association between MS calls and doctors’ prescription orders, as well as the moderating effect of MS visits on the variation in doctors’ prescription orders.

## Results

The result section describes the outcome of two analyses: the MS influence on a doctor’s new prescription behaviours and that on doctors’ behaviours following the initial prescription. The paper describes the MS impact without and with MR co-visits, followed by impact analyses by therapeutic category. More specifically, each analysis conveys four sub-analyses: 1) the MS influence without MR co-visit cases, 2) the impact by therapeutic category, 3) MS and MR joint influence, and 4) the joint impact by therapeutic category.

Table 2 highlights that, although the McNemar’s test showed a significant effect of MS calls on doctors’ prescription behaviours (p < 0.000), the Goodman-Kruskal’s gamma pointed out that the association is relatively weak in the absence of an MR joint visit (gamma = 0.2357). When MRs also contact doctors, the MS calls remain a significant predictor of doctors’ prescription behaviours concerning new drugs (p < 0.000). More in detail, the Goodman-Kruskal’s gamma showed this association to be moderately strong (gamma = 0.5981).

**Table 2.** Doctors’ responses to MS calls related to new prescriptions.

		MS influence on doctor’s prescription behavior							
		Strongly influenced		Influenced		Not influenced		Total	
MR co-visit	MR visit	1,298	(28.2%)	2,457	(53.5%)	840	(18.3%)	4,595	(100.0%)
	No MR co-visit	179	(19.2%)	491	(52.7%)	262	(28.1%)	932	(100.0%)
	Total	1,477	(26.7%)	2,948	(53.3%)	1,102	(19.9%)	5,527	(100.0%)
χ^2^ = 61.3543, p-value <0.000, Gamma = 0.2357, p-value <0.0000									
MR influence on doctor’s prescription	Strongly influenced	867	(55.4%)	537	(34.3%)	161	(10.3%)	1,565	(100.0%)
	Influenced	347	(14.8%)	1,673	(71.3%)	327	(13.9%)	2,347	(100.0%)
	Not influenced	84	(12.3%)	247	(36.2%)	352	(51.5%)	683	(100.0%)
	Total	1,298	(28.2%)	2,457	(53.5%)	840	(18.3%)	4,595	(100.0%)
χ^2^ = 1397.6459, p-value <0.000, Gamma = 0.5981, p-value <0.0000									

Note: ‘New volume’ indicates that a doctor prescribed a new drug, ‘Same volume’ indicates that a prescribing doctor continued the use of a drug at the same prescription volume, ‘Increasing volume’ indicates that a prescribing doctor increased the prescription volume of a drug, while ‘Decreasing volume’ indicates that a prescribing doctor decreased the prescription volume. ‘MR co-visit’ indicates that MR and MS jointly visited a prescribing doctor, while ‘No MR co-visits’ indicates only MS visited a prescribing doctor. Therapeutic categories were based on the MHLW classification.

The analysis regarding the MS influence on doctors’ behaviours after the initial prescription assumes three possible doctors’ behaviours: 1) maintaining the current drug volume, 2) increasing it, or 3) reducing it. The McNemar’s test showed that the MS calls exerted a significant effect on doctors’ prescription behaviours (p < 0.000), and the Goodman-Kruskal’s gamma showed this effect to be moderate in magnitude (gamma = 0.4429) in absence of MR co-visits (Table 3). It also showed that, when an MR’s influence is present, the influence of the MS calls on doctors’ prescribing behaviour is amplified (p < 0.000). More specifically, the magnitude of the effect related to combined MR/MS efforts (gamma = 0.8080) is about twice the effect when the MS only attempts to influence a doctor’s post-prescription decisions (gamma = 0.4429). As showed in Table 4, the MSs exerted a diminishing, though significant effect on a doctor’s decision to increase the prescription volume in the absence of MR calls.

**Table 3.** Doctors’ responses to MS calls for the maintaining prescription volume.

		MS influence on doctor’s prescription behavior							
		Strongly influenced		Influenced		Not influenced		Total	
MR co-visit	MR visit	4,054	(9.1%)	20,644	(46.4%)	19,810	(44.5%)	44,508	(100.0%)
	No MR co-visit	375	(3.1%)	3,544	(28.8%)	8,372	(68.1%)	12,291	(100.0%)
	Total	4,429	(7.8%)	24,188	(42.6%)	28,182	(49.6%)	56,799	(100.0%)
χ^2^ = 2231.3729, p-value <0.000, Gamma = 0.4429, p-value <0.0000									
MR influence on doctor’s prescription	Strongly influenced	2,492	(44.5%)	2,200	(39.3%)	905	(16.2%)	5,597	(100.0%)
	Influenced	1,288	(5.7%)	16,131	(70.9%)	5,327	(23.4%)	22,746	(100.0%)
	Not influenced	274	(1.7%)	2,313	(14.3%)	13,578	(84.0%)	16,165	(100.0%)
	Total	4,054	(9.1%)	20,644	(46.4%)	19,810	(44.5%)	44,508	(100.0%)
χ^2^ = 24,552.7399, p-value <0.000, Gamma = 0.8080, p-value <0.0000									

Note: ‘New volume’ indicates that a doctor prescribed a new drug, ‘Same volume’ indicates that a prescribing doctor continued the use of a drug at the same prescription volume, ‘Increasing volume’ indicates that a prescribing doctor increased the prescription volume of a drug, while ‘Decreasing volume’ indicates that a prescribing doctor decreased the prescription volume. ‘MR co-visit’ indicates that MR and MS jointly visited a prescribing doctor, while ‘No MR co-visits’ indicates only MS visited a prescribing doctor. Therapeutic categories were based on the MHLW classification.

**Table 4.** Doctors’ responses to MS calls for increasing prescription volume.

		MS influence on doctor’s prescription behavior							
		Strongly influenced		Influenced		Not influenced		Total	
MR co-visit	MR visit	2,652	(26.4%)	5,673	(56.4%)	1,737	(17.3%)	10,062	(100.0%)
	No MR co-visit	179	(15.0%)	585	(49.0%)	429	(36.0%)	1,193	(100.0%)
	Total	2,831	(25.2%)	6,258	(55.6%)	2,166	(19.2%)	11,255	(100.0%)
χ^2^ = 258.7246, p-value <0.000, Gamma = 0.3724, p-value <0.0000									
MR influence on doctor’s prescription	Strongly influenced	1,909	(50.8%)	1,473	(39.2%)	374	(10.0%)	3,756	(100.0%)
	Influenced	630	(12.2%)	3,799	(73.5%)	742	(14.3%)	5,171	(100.0%)
	Not influenced	113	(10.0%)	401	(35.3%)	621	(54.7%)	1,135	(100.0%)
	Total	2,652	(26.4%)	5,673	(56.4%)	1,737	(17.3%)	10,062	(100.0%)
χ^2^ = 2980.09, p-value <0.000, Gamma = 0.6127, p-value <0.0000									

Note: ‘New volume’ indicates that a doctor prescribed a new drug, ‘Same volume’ indicates that a prescribing doctor continued the use of a drug at the same prescription volume, ‘Increasing volume’ indicates that a prescribing doctor increased the prescription volume of a drug, while ‘Decreasing volume’ indicates that a prescribing doctor decreased the prescription volume. ‘MR co-visit’ indicates that MR and MS jointly visited a prescribing doctor, while ‘No MR co-visits’ indicates only MS visited a prescribing doctor. Therapeutic categories were based on the MHLW classification.

More than half of the doctors in the sample decided to decrease the volume of their patients’ prescriptions (Table 5). The MSs, however, affected doctors’ decisions to decrease the drug prescription volume more substantially in the absence of an MR visit (gamma = 0.8202) relative to when the MRs called doctors (gamma = 0.5106).

**Table 5.** Doctors’ responses to MS calls for decreasing prescription volume.

		MS influence on doctor’s prescription behavior							
		Strongly influenced		Influenced		Not influenced		Total	
MR co-visit	MR visit	94	(14.8%)	197	(31.1%)	343	(54.1%)	634	(100.0%)
	No MR co-visit	27	(8.0%)	74	(21.9%)	237	(70.1%)	338	(100.0%)
	Total	121	(12.4%)	271	(27.9%)	580	(59.7%)	972	(100.0%)
χ^2^ = 206.5739, p-value <0.000, Gamma = 0.8208, p-value <0.0000									
MR influence on doctor’s prescription	Strongly influenced	59	(53.6%)	31	(28.2%)	20	(18.2%)	110	(100.0%)
	Influenced	23	(10.3%)	130	(58.3%)	70	(31.4%)	223	(100.0%)
	Not influenced	12	(4.0%)	36	(12.0%)	253	(84.1%)	301	(100.0%)
	Total	94	(14.8%)	197	(31.1%)	343	(54.1%)	634	(100.0%)
χ^2^ = 76.6083, p-value <0.000, Gamma = 0.5106, p-value <0.0000									

Note: ‘New volume’ indicates that a doctor prescribed a new drug, ‘Same volume’ indicates that a prescribing doctor continued the use of a drug at the same prescription volume, ‘Increasing volume’ indicates that a prescribing doctor increased the prescription volume of a drug, while ‘Decreasing volume’ indicates that a prescribing doctor decreased the prescription volume. ‘MR co-visit’ indicates that MR and MS jointly visited a prescribing doctor, while ‘No MR co-visits’ indicates only MS visited a prescribing doctor. Therapeutic categories were based on the MHLW classification.

Table 6 illustrates the results of the analyses in which the number of calls is distinguished by therapeutic category. In this respect, the MS calls significantly influenced doctors’ prescription orders for new drugs in four out of ten categories (p < 0.05), particularly for prescriptions related to metabolism (gamma = 0.7850) and peptic ulcers (gamma = 0.7487). With MR co-visits, the MS calls significantly influenced doctors’ prescription orders for new drugs for all categories (p < 0.01), especially for antidiabetic (gamma = 0.6916) and allergic agents’ prescriptions (gamma = 0.6759).

**Table 6.** Doctors’ responses to MS calls by therapeutic category.

	New volume				Same volume				Increasing volume				Decreasing volume			
	No MR co-visits		With MR co-visits		No MR co-visits		With MR co-visits		No MR co-visits		With MR co-visits		No MR co-visits		With MR co-visits	
	X^2^	Gamma	X^2^	Gamma	X^2^	Gamma	X^2^	Gamma	X^2^	Gamma	X^2^	Gamma	X^2^	Gamma	X^2^	Gamma
Antidiabetic agents	0.5733	−0.0032	226.3749**	0.6916**	166.6594**	0.4480**	2668.1862***	0.7866***	8.0797*	0.2326**	308.1918***	0.5356***	4.9093*	0.5471**	27.439***	0.7136***
Antihypertensive agents	9.1473**	0.3236**	136.1035**	0.6618**	213.7001**	0.4533**	2703.0455***	0.8223***	17.324**	0.2976**	452.1431***	0.6839***	5.3677*	0.4119**	81.2795***	0.8454***
Allergic agents	2.6860	0.2029	94.9541**	0.6759**	81.3948**	0.3422**	2047.0722***	0.8315***	21.8989**	0.3479**	248.8388***	0.6675***	3.5657	0.3939	25.7526***	0.8454***
Agents affecting metabolism	34.9321***	0.7850***	51.7872**	0.4656**	66.9419**	0.3625**	1696.7171***	0.8329***	20.1645**	0.5609**	176.8106***	0.6229***	0.1827	0.0558	17.5192***	0.6098*
Peptic ulcer agents	18.6140***	0.7487***	24.9976**	0.4177*	115.7451**	0.4611**	1084.2496***	0.8301***	24.0946**	0.5567**	195.2998***	0.6971***	2.2425	0.4809	16.424***	0.9434***
Hyperlipimedia agents	8.5074**	0.4369**	35.3637**	0.3572*	111.7896**	0.4888**	1008.7170***	0.8218***	1.525	0.1533	170.7518***	0.6616***	2.0447	0.3265	17.8462***	1.0000***
Central nervous system agents	6.2463**	0.2902*	57.7373**	0.6745**	67.9338**	0.4274**	923.4184***	0.8116***	21.1672**	0.6202**	92.1107***	0.5892***	3.1924	0.4085	11.3467**	0.8103***
Ophthalmic agents	2.2463	0.1874	11.1424*	0.1132	40.964**	0.3723**	707.9169***	0.7712***	11.3969**	0.0727	47.1062***	0.4472***	1.7912	0.2000	6.3400	0.4937**
Respitatory agents	5.1625*	0.3638	38.1671**	0.6145**	40.5369**	0.4540**	489.8366***	0.7273***	3.8965	0.0491	126.1975***	0.6732***	0.5091	0.0357	6.5625	0.625*
Vaccines	4.8406*	0.1718	54.0001**	0.6118**	35.6923**	0.2900**	715.9562***	0.8510***	2.4038	0.1029	46.6289***	0.5581***	4.4339	−0.600**	13.4844***	0.8387**

Note: ‘New volume’ indicates that a doctor prescribed a new drug, ‘Same volume’ indicates that a prescribing doctor continued the use of a drug at the same prescription volume, ‘Increasing volume’ indicates that a prescribing doctor increased the prescription volume of a drug, while ‘Decreasing volume’ indicates that a prescribing doctor decreased the prescription volume. ‘MR co-visit’ indicates that MR and MS jointly visited a prescribing doctor, while ‘No MR co-visits’ indicates only MS visited a prescribing doctor. Therapeutic categories were based on the MHLW classification. *: p-value <0.1, **: p-value <0.05, ***: p-value <0.001

The MS calls significantly influenced doctors’ prescription orders towards maintaining the volume in all categories (p < 0.01), but not to a great extent as all gammas were lower than 0.5. With MR co-visits, this effect was stronger, as gammas were all higher than 0.7.

The MS calls significantly influenced doctors’ prescription orders towards increasing prescription volumes for seven categories (p < 0.05), with a stronger effect for medications related to central nervous system (gamma = 0.6202). With MR co-visits, the MS calls had a strong significant effect on doctors’ prescription orders towards increasing the prescription volumes in all categories (p < 0.01), except for ophthalmic drugs (gamma = 0.4472).

Only two out of ten therapeutic categories were significantly influencing doctors’ prescription orders towards modest volume’s reduction (p < 0.05). With MR co-visits, the MS calls significantly influenced doctors’ prescription orders in seven out of ten categories (p < 0.01), especially regarding the volume’s reduction of prescriptions related to hyperlipemia (gamma = 1.0000) and peptic ulcers (gamma = 0.9434).

## Discussion

The results on the MS influence on doctor’s new prescription behaviours indicate that the MR co-visits amplify such influence, while solo MS visits have a minimal impact. One reason for this outcome is that MRs tend to provide information on their own products, while MSs carry competitors’ drugs as well. In the latter case, doctors can solicit the opinions of MSs before/after prescribing a new drug. Because there is no official formulary in Japan, doctors can prescribe drugs at their own discretion. Still, they require ample information from MRs and other media to make informed prescription decisions. When the MS visits to doctors without the MR, the MS had a strong influence on prescription of medications related to metabolism and peptic ulcers. This result may be attributable to the fact that these types of drugs have been available in the market for a long time, meaning that doctors have sufficient information to responsibly prescribe them.

In case of MS and MR joint visits, their collaborative efforts further influenced doctors, highlighting significant moderating effects on the relationship between MS calls and doctor prescription decisions. These visits exerted significant moderating effects in new prescriptions mostly regarding chronic conditions. These results were in line with the results by Anderson et al. [22].

Pharmaceutical companies may rely heavily on MSs to make calls to the entire market (particularly to doctors working in hospitals with less than 19 beds or clinics) to initiate new products’ launch. Pharmaceutical companies that deal in these specific product categories can utilize the MSs in different ways to maximize the effectiveness of drugs’ marketing.

Owing to a lack of new product launches in 2014, many recent MR and MS calls have been geared towards increasing or maintaining the volume of medications prescribed by doctors. Taken together, the results indicate that MS visits are effective means for maintaining the volume of doctors’ current therapeutic preferences. Statistical results on the maintenance of prescription volumes indicated that a moderate influence of MS in the absence of an MR co-visit, but a strong joint effect of MS calls and MR co-visits. The largest effect of the combined MS calls and MR co-visits on doctors’ maintenance of prescription volumes was for vaccines, allergy medicines, medications for peptic ulcers, and drugs meant to treat life-style diseases. This effect was less strong for ophthalmic drugs and respiratory agents. One explanation for this outcome is that, as MSs began to focus on maintaining or increasing prescription volumes (rather than introduce new drugs), MS activities were more geared towards providing doctors with feedback related to current treatment regimens. This allows pharmaceutical companies to utilize MRs more effectively.

The MR visits positively moderated the effect of MS calls on a doctor’s decision to increase prescription volumes. These results suggest that the calls by MRs and MSs influence a doctor’s decision to increase the prescription’s dosage. Although MSs can influence doctors in increasing dosages in the absence of MR co-visits, the magnitude of this effect is amplified across all 10 therapeutic categories when MRs and MSs work together. This effect may be due to the fact that, once a doctor prescribes a drug, he/she appreciates scientific information on the drug’s use to ensure appropriate treatment. Because an increase in prescription volumes addresses pharmaceutical company’s objectives, the enhanced effectiveness of MSs due to cooperation with MRs is a critical finding.

It is possible that MSs that carry information related to several drugs can provide a doctor with guidance on alternative drugs, thereby decreasing the volume of an extant prescription. The MSs with salient information about several drugs exert an influence on doctors such that they decrease the volume of prescriptions for antidiabetic and antihypertensive agents. Changes in prescription volumes for other categories (e.g. agents related to life-style diseases and peptic ulcers) were likely influenced by both MSs and MRs.

Overall, the presence of MSs strengthened MRs’ influence on doctors’ prescription outcomes, particularly with respect to the maintenance of or increase in dosage regimens. Previous research has shown a negative relationship between the number of MR visits and the quality of doctors’ prescribing decisions [23]. This result is largely consistent with the positive impact on physicians in promoteing the drug to the patients after MRs’ advice [24]. Moreover, the results suggest that pharmaceutical companies can leverage wholesalers’ sales promotion behaviours and make efficient and effective use of MRs. Specifically, the MSs affect doctors’ prescribing behaviours related to well-established therapeutic categories, including antihypertensive drugs, allergy medicines, hyperlipidaemia agents, and agents affecting metabolism. In doing so, the MSs can sustain or increase the market share. There are some cases, however, in which it may be useful for MSs to advocate for a decrease in the dosing regimen of a given drug to promote an alternative medicine. Given the unique functions of the Japanese wholesalers, pharmaceutical companies should consider using MSs to promote sales and establish a mutually beneficial relationship with wholesalers.

Although this paper offers several useful findings, it still has a few limitations. First, although our findings illustrate the influence of MSs, they do not account for the calls made prior to the actual prescription; as such, there are a number of other factors that may have influenced the prescription outcomes. Second, I neither evaluated the economic value of a call, nor compared the return on calls respectively made by MSs and MRs. Even with these limitations, however, sales promotion among Japanese pharmaceutical wholesalers seems to enhance the efficiency with which a new drug is marketed to primary care doctors. Besides these limitations, there is a lack of generalizability, sampling, and recall biases since the survey was conducted on randomly selected doctors that were able to access the website provided by the survey company without reinforcement of the timing of inputting influences by MS and MR. Moreover, the survey does not ask doctors to identify nor prioritize factors such as actual invoice price, drug information, and peer review information that might have influenced them. Other factors such as doctors’ interest on prior knowledge on a specific drug or information access should be considered.

## Conclusions

This paper has shown that Japanese wholesalers’ engagement in sales promotion influences doctors’ prescriptions behaviours, particularly after a drug is initially launched and subsequently accepted by the market. Results also demonstrated that the influence wholesalers exert on doctors with respect to prescription outcomes varies by therapeutic category. Given that the role of doctors is expected to expand, the influence of drug wholesalers on doctors can be an effective strategic target for pharmaceutical companies.
